# A Cutting Pattern Recognition Method for Shearers Based on Improved Ensemble Empirical Mode Decomposition and a Probabilistic Neural Network

**DOI:** 10.3390/s151127721

**Published:** 2015-10-30

**Authors:** Jing Xu, Zhongbin Wang, Chao Tan, Lei Si, Xinhua Liu

**Affiliations:** 1 School of Mechatronic Engineering, China University of Mining & Technology, No. 1 Daxue Road, Xuzhou 221116, China; E-Mails: xujingcumt@126.com (J.X.); tccadcumt@126.com (C.T.); sileicool@163.com (L.S.); l_xinhua_2006@126.com (X.L.); 2 School of Information and Electrical Engineering, China University of Mining & Technology, No. 1 Daxue Road, Xuzhou 221116, China

**Keywords:** cutting pattern recognition, coal mining, sound signal, Improved Ensemble Empirical Mode Decomposition, intrinsic mode function, Probabilistic Neural Network

## Abstract

In order to guarantee the stable operation of shearers and promote construction of an automatic coal mining working face, an online cutting pattern recognition method with high accuracy and speed based on Improved Ensemble Empirical Mode Decomposition (IEEMD) and Probabilistic Neural Network (PNN) is proposed. An industrial microphone is installed on the shearer and the cutting sound is collected as the recognition criterion to overcome the disadvantages of giant size, contact measurement and low identification rate of traditional detectors. To avoid end-point effects and get rid of undesirable intrinsic mode function (IMF) components in the initial signal, IEEMD is conducted on the sound. The end-point continuation based on the practical storage data is performed first to overcome the end-point effect. Next the average correlation coefficient, which is calculated by the correlation of the first IMF with others, is introduced to select essential IMFs. Then the energy and standard deviation of the reminder IMFs are extracted as features and PNN is applied to classify the cutting patterns. Finally, a simulation example, with an accuracy of 92.67%, and an industrial application prove the efficiency and correctness of the proposed method.

## 1. Introduction

Nowadays, cutting pattern recognition for shearers, which aims at determining whether the shearer is cutting coal or rock, plays an important role in increasing coal output and avoiding cutting hard rock in fully-mechanized coal mining working faces. However, due to the poor working conditions during the production process, online cutting pattern recognition is always a tough technical problem [1]. Since the 1970s, domestic and international scholars have proposed a variety of methods, with little effects on solving the problem [2]. In order to ensure the shearer works safely in the long term, manual intervention is still necessary at present [3]. In industry practice, the shearer operators judge whether it is cutting coal, the rock, or coal gripping gangue, generally through the integration of geological conditions and the shearer cutting sound [4]. Then the operators adjust the shearer according to their comprehensive judgment. In fact, the cutting sound signal has its unique advantages relative to traditional vibration and current signals, such as ease of installation and maintenance, non-contact measurement and convenience for online analysis.

The preliminary analysis on the cutting sound shows that initial signal has strongly nonlinear, non-stationary and intermittent characteristics. With signal processing methods such as Short-time Fourier Transform (STFT), Wavelet Transform (WT) and Wavelet Packet Transform (WPT) it is difficult to satisfy these conditions [5]. Empirical Mode Decomposition (EMD) was proposed by Huang *et al.*, in 1998 [6]. EMD is an adaptive method to decompose any data into a set of IMFs, which become the basis of the data. As the basis is adaptive, the basis usually offers a physically meaningful representation of the underlying processes [7]. EMD is especially suitable for non-linear and non-stationary signal processing compared to the Short-Time Fourier Transform method [8,9], and superior to the Wavelet Transform and Wavelet Packet Transform methods for intermittent signals [10]. In 2004, Ensemble Empirical Mode Decomposition was proposed by Wu *et al.*, to deal with the mode mixing problem during EMD [11]. After ten years of rapid development, EEMD is nowadays widely applied in fault diagnosis [12], vibration analysis [13], signal denoising [14], speech recognition [15], forecasting [16] and so on, although there still exist some problems during EEMD, such as end-point effects and undesirable IMF components [17].

With the rapid development of electronic techniques in recent decades, Artificial Neural Networks (ANNs) have developed as an important tool in many fields [18,19,20,21]. Probabilistic Neural Networks (PNNs), which are a significant part of ANN, were proposed by Specht [22]. Since PNNs were proposed in 1988, they have been widely applied in classification [23] and pattern recognition [24]. PNNs have a feed-forward architecture and a supervised training process similar to back propagation. Each training input pattern of a PNN is used as the connection weight to a new hidden unit instead of adjusting the input layer weights using the generalized delta rule. Training of PNNs is much faster compared to Back Propagation Neural Networks (BPNNs), and PNNs allow true incremental learning where new training data can be added at any time without requiring retraining of the entire network [25]. PNNs also have better self-adaption, self-organization and self-learning ability compared with support vector machines (SVMs) and K-nearest neighbour (KNN) [26,27].

Enlightened by the above knowledge, this paper aims to propose an online cutting pattern recognition method using the cutting sound to overcome the disadvantages of high volume, low efficiency and low reliability of traditional ways. The shearer cutting sound is decomposed by an improved EEMD method to avoid end-point effects and eliminate undesirable IMF components. Then several key feature parameters, such as the energy of the reminder IMFs and standard deviation, are extracted from the real-time cutting sound. The cutting pattern is subsequently recognized by the PNN classifier. Finally, a simulation example and an industrial application are carried out to validate the effectiveness and correctness of the proposed method, and a comprehensive comparison and discussion are conducted to demonstrate the superiority in recognition speed and accuracy.

## 2. Literature Review

Recent publications relevant to this paper are mainly concerned with two research streams: coal-rock cutting pattern recognition methods and EEMD. In this section, we try to summarize the relevant literature.

### 2.1. Coal-Rock Cutting Pattern Recognition Methods

Coal-rock cutting pattern recognition is the biggest technical bottleneck in the shearer auto-control field. To solve this problem, more than 20 kinds of approaches have been proposed. The most influential methods are γ-ray detection means [28], radar detection means [29], infrared detection means [30], image detection means [31], mechanical vibration means [32], memory cutting means [33], *etc.* The first three methods recognize the coal and the rock by installing special signal emitters and receivers, and the judgment result is obtained by analyzing the received signal. Up to now, these methods mainly focus on the theoretical field [34]. In [31], a recognition method based on image feature extraction was proposed, whereby a coal-rock image was decomposed with use of Daubechies wavelet and texture orientation degrees were structured, and the cutting pattern recognition results proved the method was efficient. In [32], a new approach based on the wavelet packet energy spectrum via the vibration signal was proposed to identify the coal-rock interface in top coal caving. Wang *et al*. [33] proposed a self-adaptive memory cutting method for shearers, where key technologies of memory cutting were studied and actual production tests show that the absolute error of the cutting path was less than 0.06 m.

### 2.2. Ensemble Empirical Mode Decomposition

Since EEMD is an adaptive method, it has an extensive applications in industry practice. While there still exists some problems in EEMD such as the end-point effect and undesirable IMF components, generally, end-point effects can be solved effectively by end-point continuation based on the practical storage data [35]. Undesirable IMF components contain redundant and contradictory IMFs, which will increase the computational computation and decrease the recognition accuracy for subsequent processing. An ideal IMF contains only single-frequency components and the same frequency components are distributed only in single-IMF [36]. To eliminate undesirable IMF components, many approaches were proposed. In [17], a modified EEMD was presented to achieve more accurate and reliable sensor data. By comparing the threshold value and correlation coefficients between IMFs and the initial signal, it could be determined whether the IMF should be retained or not, then the signal was reconstructed by the remainder IMFs. Jiang *et al*. [37] proposed a novel approach of condition monitoring and fault diagnosis for rolling element bearings based on an improved EEMD. The primordial signal was decomposed by the improved EEMD, key components were reserved and then the correlation analysis was introduced to extract statistical features. In [38], a novel bearing fault diagnosis method based on EEMD and the Teager energy operator was proposed. The EEMD was firstly applied to obtain monocomponents, then the IMF of interest was selected according to its correlation with original signal and its kurtosis, and the Teager energy operator was applied to detect fault-including periodic impulses. Two years later, Yi *et al*. [39] introduced IMF confidence index arithmetic to overcome the limitation of needing users with experience to select desirable IMFs. The index consisted of self-correlation quality, absolute skewness, kurtosis and impact allowance.

### 2.3. Discussion

Although many cutting pattern recognition methods have been developed, they share some common disadvantages. Firstly, the coal-rock detectors used in the prior literature are complex and bulky, which cannot satisfy the needs for wide application in practical production. The recognition rate is specially influenced under the conditions of coal seam gripping gangue. Moreover, the shearer operators take the cutting sound as an important measure to distinguish the coal and the rock during coal mining, but few are engaged in the related academic research.

There also exist the problems of end-point effects and undesirable IMF components during normal EEMD. Some scholars select IMFs though the correlation of IMF components and the initial signal, but the correlation between the IMFs is rarely studied. Moreover, the first IMF during EEMD always contains much key information, but the correlation coefficients between IMF1 and others have also rarely been researched.

Previously, the authors have carried out much research on memory cutting and coal seam terrain prediction through intelligent algorithms such as D-S evidence theory, neural network and artificial immune algorithms, although in practice the effect is unsatisfactory, as the geological conditions of the coal seam are always changing with the mining production and frequent manual intervention is still inevitable in practical application. Moreover, the relationship between the cutting sound and its corresponding pattern has never been studied in depth. In this paper, the shearer cutting sound is collected as the cutting pattern recognition criterion. In order to avoid end-point effects and eliminate undesirable IMF components, an improved EEMD is proposed. The end-point continuation based on the practical storage data is implemented first, and correlation coefficients between the first IMF and others are calculated to select essential IMFs. Then the energy and standard deviation of the selected IMFs are extracted as the feature vector. Finally, PNN is utilized as the classifier to realize the cutting pattern recognition.

## 3. The Proposed Method

### 3.1. Improved Ensemble Empirical Mode Decomposition

In order to eliminate the mode mixing problem, EEMD was put forward on the basis of EMD. According to Huang *et al.*, the key of EMD and EEMD is the concept of IMF, which contains the local information embedded in the original signal. Any complex time series can be decomposed into several IMFs and a residue component through the decomposition. The IMF must satisfy the following two conditions:

(a) The number of extremes and number of zero crossings must either be equal or differ at most by one.

(b) The mean value of the envelopes defined by the local maxima and minima is zero at any point.

Based on the principles of IMF, the algorithm of EEMD is given as below:

*Step 1.1*:  Add a random white noise *y_i_*(*t*) to the original signal series *X*(*t*):
  (1) $$X_{i}(t)=X(t)+y_{i}(t)$$
  where *X_i_*(*t*) is the noisy-added signal, *i* = 1, 2… *k*, and *k* is the number of attempts.
*Step 1.2*:  For the noisy-added signal *X_i_*(*t*), all extrema are searched at first. The upper and the lower envelopes are respectively constructed by connecting all the maxima and the minima through cubic splines. The mean of the two envelopes is defined as $m_{1}^{i}$, then subtract the $m_{1}^{i}$ from *X_i_*(*t*) to get a component $h_{1}^{i}$, which can be described as follows:
  (2) $$h_{1}^{i}=X_{i}(t)-m_{1}^{i}$$
  If $h_{1}^{i}$ satisfies the two conditions of IMF, then $h_{1}^{i}$ is the first IMF of *X_i_*(*t*), called $C_{1}^{i}$. Else $h_{1}^{i}$ is treated as the original signal *X_i_*(*t*) and repeat the above step. Generally, $C_{1}^{i}$ contains the highest frequency component of the signal.
*Step 1.3*:  Separate $C_{1}^{i}$ from *X_i_*(*t*), the remainder $r_{1}^{i}$ can be defined as follows:
  (3) $$r_{1}^{i}=X_{i}(t)-C_{1}^{i}$$
  Then *X_i_*(*t*) is replaced by $r_{1}^{i}$, repeat the above operation until *N_i_*-th remainder, namely $r_{Ni}^{i}$, becomes a monotonic function. $r_{Ni}^{i}$ can be expressed as follows:
  (4) $$r_{N_{i}}^{i}=r_{N_{i}-1}^{i}-C_{N_{i}}^{i}$$
*Step 1.4*:  *X_i_*(*t*) can be decomposed by the sum of *N_i_* IMFs and a residual, which can be shown as follows:
  (5) $$X_{i}(t)=\sum_{j=1}^{N_{i}}{C^{i}}_{j}+r_{N_{i}}^{i},\;i=1,2,3...k$$
  where $r_{Ni}^{i}$ is the residual, represents average trend of *X_i_*(*t*).
*Step 1.5*:  Calculate *N* = min{*N*_1_, *N*_2_… *N_k_*} and the ensemble means of corresponding IMFs of the decomposition as the final result:
  (6) $$C_{n}=(\sum_{i=1}^{k}C_{n}^{i})/k,\;n=1,2,3...N$$
  where *C_n_*(*n* = 1,2…*N*) is the ensemble means of corresponding IMFs of the decomposition. In addition, the number of attempts is 100 and the standard deviation of the added noisy is 0.2 times that of the original signal, as suggested by Wu and Huang. In practical use, EEMD has a better effect than EMD. In this paper, an improved EEMD is presented to settle the problems of end-point effect and undesirable IMF components, which can be elaborated as follows:
*Step 2.1*:  The redundancy threshold ξ is introduced at first. *M* data series are selected and the length of each sequence is set as *L*. Based on the original storage data, extension is operated on the left and right of the data series with the length of *l* respectively. So the length of each analytic data is computed as *L* + 2*l*.
*Step 2.2*:  The data series are decomposed by EEMD to obtain a suite of IMFs. Considering the number of IMF may differ from each other, the biggest number is selected as *T*_max_. If the IMF number is smaller than *T*_max_, some zero vectors are supplemented in low frequency.
*Step 2.3*:  The extended data on both sides of every IMF are eliminated, so the result of EEMD can be expressed as follows:
  (7) $$X_{m}=\sum_{t=1}^{T_{\max}}IMF_{m,t}+re_{m},\;m=1,2,3...M$$
  where *X_m_* is *m*-th series, IMF*_m_*_,*t*_ is *t*-th IMF of *m*-th series, *re_m_* represents the residual, and the length of *X_m_* is *L*.
*Step 2.4*:  IMF1 is set as the first IMF, then correlation coefficients of IMF1 and IMF2, IMF1 and IMF3, ..., IMF1 and IMF*T* are calculated. The average correlation coefficient named *r_t_* is introduced and can be defined as follows:
  (8) $$r_{t}=\left(\sum_{m=1}^{M}\left|corcoe_{t}^{m}\right|\right)/M,\;t=2,3...T$$
  where $corcoe_{t}^{m}$ represents the correlation coefficient between IMF*t* and IMF1 of *m*-th series.
*Step 2.5*:  The average correlation coefficients with the redundancy threshold ξ are compared and the coefficients greater than ξ are deleted. Also, IMFs correspond to the deleted coefficients are removed and the number of reminder IMFs can be marked as *T*.

**Figure 1:**
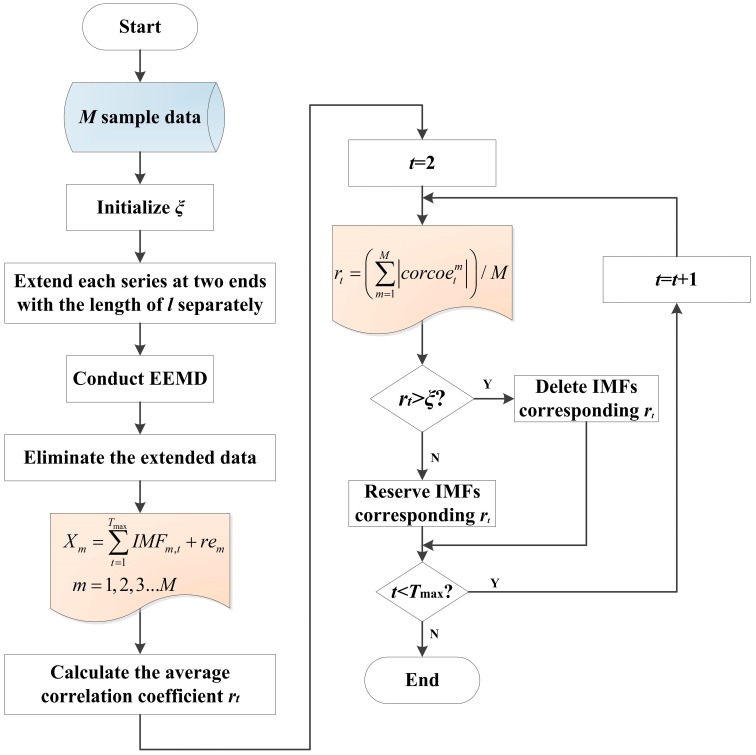
Flowchart of improved EEMD.

Obviously, the key point of the improved EEMD is choosing an appropriate redundancy threshold ξ, the flowchart of the proposed process can be shown in Figure 1.

### 3.2. Feature Extraction

In order to extract critical features from the IMFs decomposed by the EEMD, the energy *E_t_* and the standard deviation *SD_t_* are calculated as follows:

(9) $$E_{t}=\sum_{l=1}^{L}x_{l}^{2}，\;SD_{t}=\sqrt{\frac{1}{L}({\sum_{l=1}^{L}\left(x_{l}-\bar{x}\right)}^{2})}$$

where *E_t_* represents the energy of *t-*th IMF, *SD_t_* is the standard deviation, *L* is the length of the IMF, *x_l_* is *l*-th point of the IMF, $\bar{x}$ is the average value and the number of IMF is *T*.

Then the feature vector *Y* is generated with the normalized energy and normalized standard deviation as the elements:

(10) $$Y=\left\{E_{N1},SD_{N1},E_{N2},SD_{N2}...E_{Nt},SD_{Nt}...E_{NT},SD_{NT}\right\}$$

where *E_Nt_* stands for the normalized energy of *t*-th IMF, and *SD_Nt_* represents the normalized standard deviation.

### 3.3. Probabilistic Neural Network

The Probabilistic Neural Network (PNN) is a supervised feed-forward neural network that is widely applied in the field of pattern recognition. A typical PNN contains input layer, model layer, summation layer and output layer. It is assumed that a *d*-dimensional input vector ***x*** could be expressed as ***x*** = [*x*_1_, *x*_2_… *x_d_*]*^T^*, and the vector can be classified into one of the *c* categories(ω_1_, ω_2_… ω*_c_*). The neuron number of input layer is identical to the dimension of ***x***, the input layer takes charge of receiving and transporting the test samples. There exist *c* groups of neurons in the model layer, where each group corresponds to a category. The neuron number of each group is equal to the test sample amount of the corresponding category, and each neuron connects to the input layer completely. The output of *j*-th neuron in *i*-th group can be expressed as follows:

(11) $$\phi_{ij}(x;\sigma )=\frac{1}{{\left(2\pi \right)}^{d/2}\sigma^{d}}\exp[-\frac{{\left(x-x_{i}^{j}\right)}^{T}(x-x_{i}^{j})}{2\sigma^{2}}]$$

where *i* = 1, 2…*c*, *j* = 1, 2… *N_i_*, *N_i_* is the sample number of *i*-th group, σ is the smooth factor, $x_{i}^{j}$ is *j*-th training sample of category ω*_i_*. In summation layer, there are *c* nodes, and each node connects to the homologous neuron in model layer. The result of *i*-th node can be calculated as follows:

(12) $$f_{i}(x;\sigma )=\frac{1}{N}\sum_{j=1}^{N_{i}}\phi_{ij}(x;\sigma )$$

The final output of each model in output layer can be expressed as follows:

(13) $$O_{i}=P(\omega_{i})\times f_{i}(x;\sigma )$$

where *P*(ω*_i_*) is the prior probability of *i*-th category. For the input vector ***x***, if *i ≠ j* (*i*, *j* ∈ [1, 2, 3… c]) and *O_i_* > *O_j_*, then the vector can be classified into ω*_i_*.

### 3.4. Processing Method Based on IEEMD and PNN

**Figure 2:**
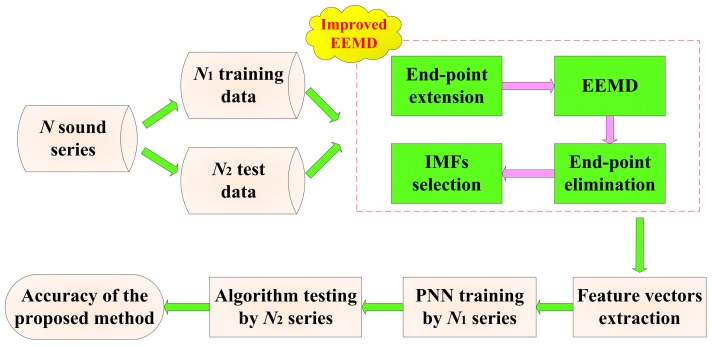
Flowchart of the cutting pattern recognition method based on IEEMD and PNN.

According to the end-point extension and average correlation coefficients, an online cutting pattern recognition approach through the cutting sound based on IEEMD and PNN can be presented as follows:

(1) Acquire *N* sample sound series in different cutting patterns and divide them into *N*_1_ training data and *N*_2_ testing data.
(2) Extend the *N* series and decompose them into several IMF components. Then eliminate the continuation data and select essential IMF components. The selection process is confirmed by the relationship between the average correlation coefficients and the redundancy threshold ξ.
(3) Extract the energy and standard deviation of the reminder IMFs as features, and normalize the feature vector of the sound series. Input the extracted vectors of *N*_1_ training series into the initial PNN, and the cutting pattern is the output of PNN.
(4) Input the feature vectors of the testing series into the trained PNN, and acquire the cutting pattern of each testing sample finally. The flowchart of cutting pattern recognition method can be shown in Figure 2.

## 4. Simulation and Analysis

In this section, a simulation example is put forward to verify the efficiency and correctness of the proposed method. Sound of five different kinds of cutting pattern was collected, respectively. Then IEEMD, feature extraction and PNN classification processing were performed in order. Some comparison and analysis were conducted according to the simulation example.

### 4.1. Sample Data Acquisition

**Figure 3:**
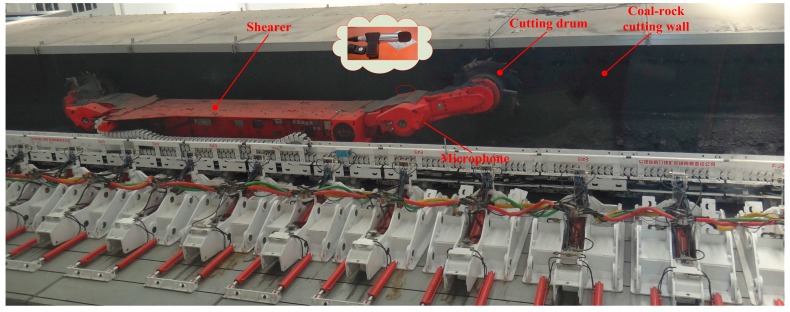
The experimental site.

The sample data were acquired from the National Coal Mining Equipment Research and Experiment Center at the China Coal Zhangjiakou Coal Mining Machinery Co., Ltd. A 70 m long cutting wall, with the height of 1.2 m, was built to simulate real geological conditions, and the shearer model was an MG500/1130-WD. The cutting wall contained four sections: a 20 m long pure coal seam with a Protodikonov hardness coefficient of f2 (C1), a 20 m long pure coal seam with a hardness of f3 (C2), a 15 m long pure hard rock seam (C3) and a 15 m long coal seam including rocks (C4). The traction speed of the shearer was a constant 3 m/min and the cutting drum rotate speed was 25 r/min. An industrial microphone was utilized to record the cutting sound of C1, C2, C3, C4 and the condition with no-load (C5), as shown in Figure 3. The sampling frequency of the sound signals was 44.1 kHz and 600 sample series, each with a duration time of 0.5 s (*L* = 22.05 × 10^3^) were collected. Half (300 series) were treated as the training samples and the remaining 300 series were the testing samples.

### 4.2. Sound Decomposition and Feature Extraction

**Figure 4:**
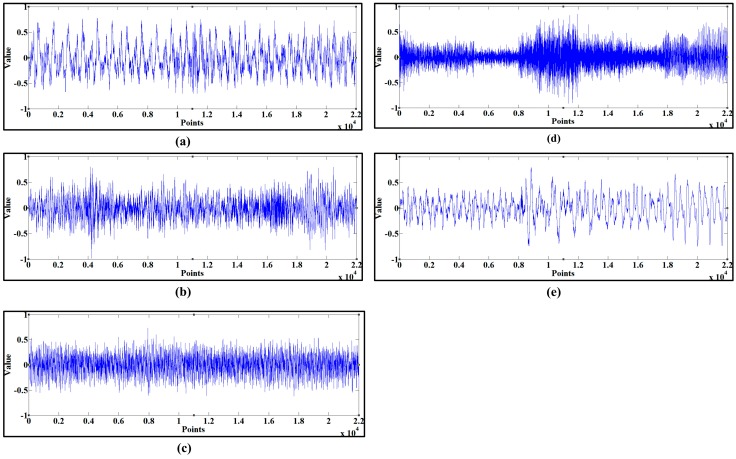
The initial sound series of the five patterns. (**a**) pure coal seam with the hardness of f2; (**b**) pure coal seam with the hardness of f3; (**c**) pure hard rock seam; (**d**) coal seam gripping gangue; (**e**) no-load.

**Figure 5:**
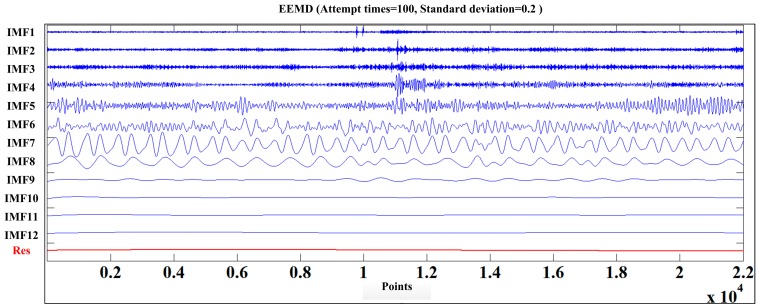
The decomposition result of C1.

The cutting sound of the shearer for different cutting patterns was decomposed according to the IEEMD. The initial data series of the training samples is shown in Figure 4. Extension was first applied the initial series and *l* was set as 100. Then the standard EEMD was conducted and the extended parts were eliminated subsequently, where the result of C1 is shown in Figure 5.

After decomposing 600 sound series, the biggest IMF component number *T*_max_ was 14, so an arbitrary signal *X_m_* can be described by Equation (14) and zero vectors were supplemented in low frequency for series without *T*_max_ IMFs.

(14) $$X_{m}=\sum_{t=1}^{14}IMF_{m,t}+re_{m}$$

where *IMF_m,t_* was *t*-th IMF component of *m*-th sound series, *m* = 1, 2, 3... 600.

The average correlation coefficients of the training series were calculated and are listed in Table 1. The preliminary redundant threshold ξ was 0.1 times the biggest average correlation coefficient, so the threshold value was 0.0024. The remainder IMF components were IMF1, IMF2, IMF4, IMF6, IMF7, IMF9, IMF10 and IMF13.

**Table 1:** The average correlation coefficients of the training samples.

*r*_1_	*r*_2_	*r*_3_	*r*_4_	*r*_5_	*r*_6_	*r*_7_	*r*_8_	*r*_9_	*r*_10_	*r*_11_	*r*_12_	*r*_13_
0.0020	0.0029	0.0016	0.0035	0.0007	0.0012	0.0047	0.0021	0.0020	0.0031	0.0240	0.0019	0.0075

The IEEMD eliminated undesirable IMF components from the original sound signal according to the redundant threshold ξ. The energy and standard deviation of the remainder IMFs were extracted as the features according to Equation (9). In order to facilitate computation and training of the PNN, normalization was operated subsequently. For an arbitrary *x_i_* ∈ [*b*, *a*], the normalization process could be shown as follows:

(15) $$x_{Ni}=\frac{x_{i}-a}{b-a}$$

where *b* was maximum value of *x_i_*, *a* was minimum value and *x_Ni_* was the normalized value of *x_i_*.

After normalizing the energy and standard deviation of the eight IMFs, a 16-dimensional vector was obtained according to Equation (10). The 16 elements were organized as the feature vectors representing each sound series and all 600 feature vectors are shown in Table 2.

**Table 2:** Feature vectors of the 600 sound samples.

Training Sample Number	Feature Vector
1	[0.0672, 0.1010, 0.3673, 0.0809, 0.6901, 0.1071, 0.7625, 0.946, 0.9218, 0.3012, 0.0362, 0.0421, 0.0043, 0.056, 0.0026, 0.0192]
2	[0.7037, 0.1662, 0.8445, 0.1760, 0.2710, 0.3091, 0.7370, 0.2522, 0.3111, 0.1631, 0.0063, 0.0172, 0.0353, 0.0132, 0.0084, 0.0006]
3	[0.9808, 0.0153, 0.0395, 0.3762, 0.6742, 0.0559, 0.7328, 0.0186, 0.6364, 0.0138, 0.1120, 0.0022, 0.5197, 0.8962, 0.58806, 0.0015]
......	
599	[0.0650, 0.0163, 0.3948, 0.0138, 0.1327, 0.0096, 0.2402, 0.0033, 0.6132, 0.7146, 0.3410, 0.0004, 0.0578, 0.0053, 0.0297, 0.0307]
600	[0.0118, 0.0023, 0.03407, 0.0038, 0.1841, 0.0087, 0.7196, 0.0622, 0.0343, 0.0566, 0.5617, 0.0059, 0.2671, 0.0036, 0.0644, 0.0016]

### 4.3. PNN Training and Testing

**Figure 6:**
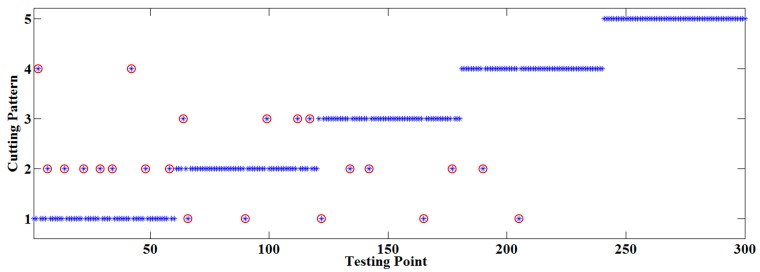
Comparison between actual pattern and PNN prediction result.

The initial PNN was trained with the 300 training series, where the inputs of the network were the extracted vectors and the outputs were the corresponding cutting patterns. The node number of the input layer was 16, and there were five groups of nodes in the model layer, where each group contained 60 training samples. Obviously, five nodes corresponding to the cutting patterns existed in the summation layer, and the output of the PNN was one of C1, C2, C3, C4 and C5. The smoothing factor σ in the PNN was 0.1, the prior probability of each category was 1/5 in the model layer and the iteration number was set as 1000. Then testing samples were treated by the trained PNN and comparison was made between the actual cutting pattern and PNN prediction results, as shown in Figure 6. Twenty two misjudgments occurred in the 300 testing samples so the simulation accuracy was 92.67%.

Seen from Figure 6, C1 and C2 were misidentified nine times, C2 and C3 were misjudged seven times, C1 and C4 were confused three times, C1 and C3 were mistaken two times and C4 was classified as C2 once. The reason lies in the fact the sound of cutting objects with similar hardness had small differences, and those with obvious distinction could be recognized accurately.

Finally, to obtain the change rule of the reminder IMF number and the prediction accuracy at different redundancies, seven thresholds were selected and compared according to the distribution rule of the average correlation coefficients, and the results are shown in Table 3.

**Table 3:** Change rule of the reminder IMF number and the prediction accuracy at different ξ.

ξ	Reminder IMF Number	Dimension of Feature Vector	Simulation Accuracy
0.05	3	6	47.33%
0.08	5	10	73.00%
0.10	8	16	92.67%
0.15	11	22	83.33%
0.20	12	24	92.00%
0.35	13	26	83.67%
1.00	14	28	85.00%

In Table 3, the reminder IMF number was increased along with ξ, and the simulation accuracy was firstly increasing and then vibrating. Moreover, two peak values appear at ξ = 0.1 and ξ = 0.2 respectively, the former being slightly bigger than the second. Obviously, the computational complexity will increase with ξ, which means more elements are extracted as the PNN input nodes, so 0.1 can be treated as the optimum value.

### 4.4. Discussion

In order to demonstrate the superiority of the proposed method to those proposed by other similar researchers, a comparison and analysis was presented in this paper. According to the relevant literature, natural γ-ray detection, WPT and PNN, traditional EEMD and PNN, and the improved method in [17] were selected as representative approaches to make a comparison to the IEEMD and PNN. The recognition accuracy of the 300 testing samples and recognition time were treated as evaluation criteria, and the results are shown in Table 4. Limited by the poor working conditions, natural γ-rays and WPT showed poor performance, both in recognition accuracy and time. It is necessary to point out that any electrical equipment used at a coal mining face must satisfy the explosion-proof standard and consequently a special structure was needed, which resulted in the volume of the γ-ray detector being about 25 times that of the microphone. As the sound signal was strongly non-stationary, it was difficult for WPT to extract key information due to the fixed wavelet function and decomposition layer. On the other hand traditional EEMD and Yu’s methods were obvious improvements compared to the former approaches. The reason lies in that the EEMD have complete self-adaptiveness and local ability, both in physical space and frequency space. Moreover, Yu’s method eliminated some undesirable IMFs, and the recognition time decreased significantly. Compared with the above approaches, the proposed method removed undesirable IMFs and retrained essential ones to the maximum degree. In summary, the proposed IEEMD and PNN had the best comprehensive performance in the comparison.

**Table 4:** Comprehensive performance of related methods.

Compared Methods	Reminder IMF Number	Recognition Accuracy	Recognition Time (s)
Natural γ-ray detection	—	66.67%	92.7469
WPT and PNN	—	78.33%	65.0264
Traditional EEMD and PNN	14	86.00%	50.3133
Yu’s method	9	87.67%	46.1962
The proposed method	8	92.67%	45.0917

## 5. Industrial Application

**Figure 7:**
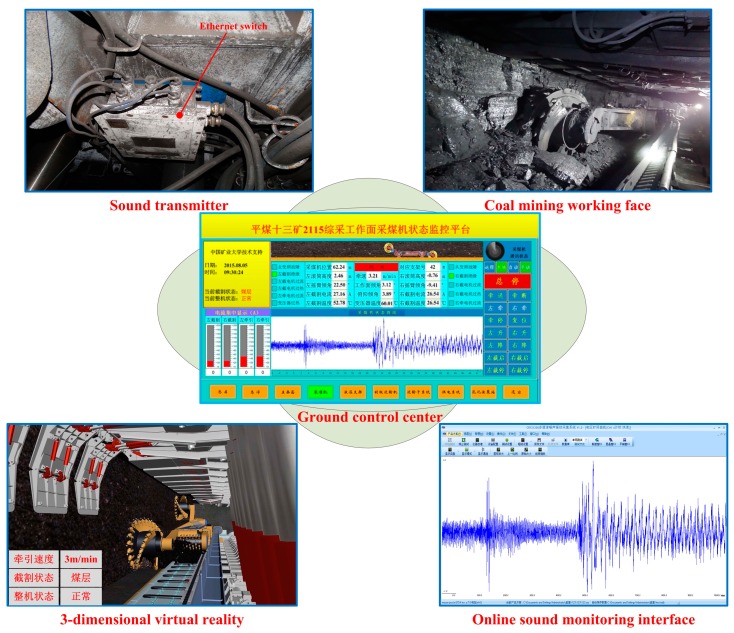
Industrial application of the proposed method.

**Figure 8:**
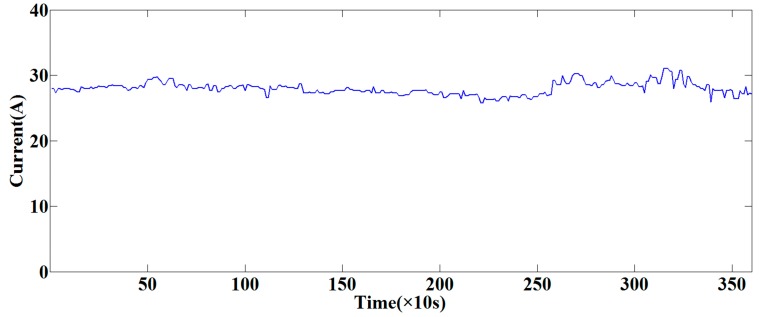
Left cutting current curve in an hour.

In this section, an online system based on the proposed method had been developed and applied in the field of automatic coal mining as shown in Figure 7. The application was tested at the 2115 coal mining face in the No. 13 Mine of the Pingdingshan Coal Industrial Group Corporation. An explosion-proof microphone was installed to collect the cutting sound, and the sound was transformed by an Ethernet switch. The ground monitoring and control platform consisted of a ground control center, a 3-dimensional virtual reality platform and an online sound monitoring interface. In order to illustrate the effectiveness of the contrasted system, the left cutting current of the shearer was collected and the curve is shown in Figure 8. The current data was from 19:26:00 to 20:26:00 on 10 August 2015. As seen from Figure 8, the left cutting current was changed in the scope of 25.7716 A to 31.0185 A as the time elapsed, and the average value was 27.9600 A. The maximum current was only about 10.9388% larger than that of the average value. The cutting load was uniform and the occurrence of cutting rock was avoided, which proved the stability and reliability of the online system.

## 6. Conclusions and Future Work

In order to realize online cutting pattern recognition during coal mining, this paper proposes a novel approach using the cutting sound based on an improved EEMD and PNN. Improved strategies on the basis of end-point continuation and correlation of IMF1 with other IMF components were applied in EEMD to avoid end-point effects and eliminate undesirable IMF components, and then PNN was used as the classification algorithm. To verify the feasibility and superiority of the proposed approach, a simulation example was provided and some comparisons were conducted. The simulation example and comparison results showed that the online cutting pattern recognition method could effectively distinguish the cutting pattern and the proposed approach outperformed others.

However, there are also some limitations and bugs in this method that may be listed as follows: (1) The redundant threshold of the average correlation coefficient is selected by extensive simulations. Strict mathematical derivation is absent in the process, which increases blindness of the recognition system; (2) the traction speed of the shearer is a constant 3 m/min, which does not match the changing practical conditions; (3) the online system still has the problem of response delay in the present model. In future studies, the authors plan to investigate some improvements to the proposed approach. These may include an adaptive algorithm to select an appropriate redundant threshold, a cutting pattern recognition method for changing cutting speeds and higher execution efficiency of the algorithm code.
